# A Scoping Review of Interactions between Omega-3 Long-Chain Polyunsaturated Fatty Acids and Genetic Variation in Relation to Cancer Risk

**DOI:** 10.3390/nu12061647

**Published:** 2020-06-02

**Authors:** Karin Yurko-Mauro, Mary Van Elswyk, Lynn Teo

**Affiliations:** 1 Pharma Segment, DSM Nutritional Products, Columbia, MD 21045, USA; 2 Van Elswyk Consulting, Inc., Clark, CO 80428, USA; mveconsulting@q.com; 3 Teo Research Consulting, Silver Spring, MD, 20910, USA; teoresearchconsulting@gmail.com

**Keywords:** polyunsaturated fatty acids, omega-3, docosahexaenoic acid, eicosapentaenoic acid, cancer, genes, genetic variation, genotype, scoping review

## Abstract

This scoping review examines the interaction of docosahexaenoic acid (DHA) and eicosapentaenoic acid (EPA) and genetic variants of various types of cancers. A comprehensive search was performed to identify controlled and observational studies conducted through August 2017. Eighteen unique studies were included: breast cancer (*n* = 2), gastric cancer (*n* = 1), exocrine pancreatic cancer (*n* = 1), chronic lymphocytic leukemia (*n* = 1), prostate cancer (*n* = 7) and colorectal cancer (*n* = 6). An additional 13 studies that focused on fish intake or at-risk populations were summarized to increase readers’ understanding of the topic based on this review, DHA and EPA interact with certain genetic variants to decrease breast, colorectal and prostate cancer risk, although data was limited and identified polymorphisms were heterogeneous. The evidence to date demonstrates that omega-3 long-chain polyunsaturated fatty acids (n-3 LC-PUFA) may decrease cancer risk by affecting genetic variants of inflammatory pathways, oxidative stress and tumor apoptosis. Collectively, data supports the notion that once a genetic variant is identified, the benefits of a targeted, personalized therapeutic regimen that includes DHA and/or EPA should be considered.

## 1. Introduction

Cancer is a growing global burden. In 2018, there was an estimated 18.1 million cancer cases and 9.6 million cancer deaths worldwide [1]. Globally, the leading types of cancer among men are lung, prostate and stomach cancers that represent 14.5%, 13.5% and 7.2% of all new cases, respectively. In women, breast, lung and cervical cancers are the leading cancer types with 24.2%, 8.4% and 6.6% of new cases, respectively. [1]. According to a National Vital Statistic Report released in 2019, cancer was the second leading cause of death (exceeded by heart disease) and 21.4% of deaths in the US were due to cancer in 2017 [2]. While considerable progress has been made in the diagnosis and treatment of many cancers, standard treatments not only cause apoptosis of the cancer cells, but also surrounding healthy cells and tissues. This leads to weakened and dysfunctional tissue, and contributes to adverse events, such as fatigue, nausea, muscle loss, diarrhea or constipation. Such side effects can be severe and lead to dose reductions or the discontinuation of the drug treatment. More tailored, personalized medicine and nutritional approaches based on genetics, drug responsiveness, and immune function, along with an optimal diet are being advanced as therapies for cancer treatment.

Omega-3 long-chain polyunsaturated fatty acids (n-3 LC-PUFA), such docosahexaenoic acid (DHA) and eicosapentaenoic acid (EPA), are important fatty acids that may play a role in preventing some cancers and act as adjunct therapy to chemotherapeutics, immunotherapeutics, or radiation [3]. While the n-3 LC-PUFA precursor, alpha-linolenic acid (ALA), is often more prevalent in the diet than DHA and EPA, it is recognized that the conversion of ALA to downstream metabolites is limited and considered insufficient to support tissue n-3 LC-PUFA levels that are associated with various biological outcomes [4]. The same is true for the production and intake of intermediate n-3 LC-PUFA, such as docosapentaenoic acid (DPA), the effects and mechanisms of action of which are poorly understood [4]. With regard to cancer risk in particular, evidence suggests a limited role of ALA in reducing development of various tumor types, yet greater biopotency of preformed DHA and EPA in a variety of pathways related to cancer risk reduction [5,6,7].

N-3 LC-PUFA have pleiotropic effects and enhance cancer cell apoptosis, modulate various eicosanoid pathways leading to reduced inflammation, such as suppressing cyclooxygenase-2 (COX-2) synthesis and the inhibition of arachidonic acid-derived eicosanoids [8]. Importantly, N-3 LC-PUFA’ anti-neoplastic activity may derive from their incorporation into cell membranes to optimize receptor function and signaling pathways, including inhibiting Ras/ERK pathway and phosphoinositide signaling (e.g., AKT inactivation) [9,10] which may arrest cancer cell growth. Their ability to increase intracellular oxidative stress [11] and to bind to nuclear receptors to modulate gene expression paths of apoptosis [12] are also potential anti-cancer mechanisms. Animal studies and human observational studies have demonstrated that n-3 LC-PUFA may reduce the risk of cancers such as breast, colon and prostate [8,10,13]. The effects of n-3 LC-PUFA, however, may be modulated by specific genes or genetic variants of cancer phenotypes leading to variations in therapeutic response [14]. For example, a case-control study by Fradet et al. (2009) found that greater intake of n-3 LC-PUFA was significantly associated with lower prostate cancer (PCA) risk in those with the cyclooxygenase-2 (COX-2) single nucleotide polymorphism (SNP) rs4648310 [15]. While some other studies have demonstrated similar positive effects of n-3 LC-PUFA on specific genetic variants of cancer [11,16], other studies have had null or mixed results [17,18] and many studies have not explored such genotype-dependent effects. 

This scoping review was undertaken to identify human studies that examined the effects of preformed n-3 LC-PUFA, namely DHA and EPA, on genetic variants of any type of cancer. Both observational and interventional studies are included. The goal is to better understand associations between preformed n-3 LC-PUFA intake or tissue levels and genotype-specific cancer risk, and to understand variability in individual responses to n-3 LC-PUFA supplementation in order to provide a targeted, personalized therapeutic approach to cancer treatment.

## 2. Materials and Methods

### 2.1. Study Inclusion and Exclusion Criteria

Human studies were included if they met the following criteria: (1) peer-reviewed publications of trials, including controlled trials (randomized or non-randomized) or observational trials; (2) involving healthy subjects or cancer patients; where (3) n-3 LC-PUFA (DHA and/or EPA) were given as an intervention or DHA and/or EPA blood or tissue levels were measured or the intake of DHA and/or EPA was reported; (4) and the association with risk of cancer by genotype or expression of genes involved in carcinogenesis was measured. All types of control/comparators were considered for inclusion.

Case reports, protocols, conference abstracts, letters, commentaries as well as animal or cell-line studies were excluded. Studies in which the independent effects of DHA and/or EPA could not be isolated due to combination with another active ingredient or not separately analyzed in the diet or tissue from other n-3 LC-PUFA (e.g., alpha-linolenic acid, docosapentaenoic acid, etc.) were also excluded. 

### 2.2. Data Sources and Search Strategy

**Figure 1:**
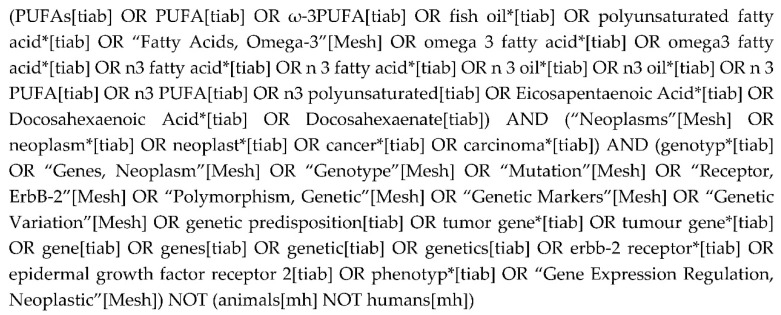
PubMed search string.

PubMed, Embase, Scopus, World of Science and Cochrane’s CENTRAL were searched from their database inception through August 2017. All searches were restricted to the peer-reviewed studies involving human subjects. See Figure 1 for the PubMed search string that was executed. The variations used for other databases can be accessed by contacting the corresponding author. Systematic reviews, meta-analyses and Clinicaltrials.gov were also scanned for salient references.

### 2.3. Study Selection

Two investigators (L.T. and M.V.E.) independently screened titles and abstracts of all citations yielded from the literature search in duplicate using the pre-defined study eligibility criteria. Conflicts regarding inclusion of citations were resolved by consensus between the two investigators and when consensus could not be reached, a third party was consulted (K.Y.M). Full texts were acquired for any abstract meeting all eligibility criteria or for those where eligibility could not be determined. Full-texts of abstracts reporting only fish intake were acquired to see if DHA or EPA intake or tissue levels were reported. If not reported in the full text, authors of these studies were contacted; contacted authors either did not respond or replied that these levels were not measured. During this process, recognizing the limited data available for preformed DHA and EPA, the authors elected to summarize and include in the evidence map, studies reporting only fish intake since fish is the primary source of EPA and DHA. 

Studies reporting only fish intake as well as studies reporting estrogen receptor (ER) status or progesterone receptor (PR) status of breast cancer tumors, and studies involving participants with familial adenomatous polyposis (FAP), were summarized to provide additional insights into those meeting the inclusion criteria. FAP is an inherited disorder associated with mutations in the adenomatous polyposis coli (APC) gene and colorectal cancer (CRC). Thus, FAP patients are particularly at risk for developing CRC.

ER+ is the most common breast cancer with 2 out of 3 cases being hormone receptor positive, and most of these ER+. The BReast CAncer susceptibility gene (BRCA) is a genetic mutation predisposing one to an increased risk of breast and/or ovarian cancer. Generally, BRCA 2 tumors are ER+. Given the relationship between ER+ and BRCA 2, it was of interest to see if any studies report associations with BRCA or ER status and n-3 LC-PUFA in blood or diet.

## 3. Results

### 3.1. Study Selection

**Figure 2:**
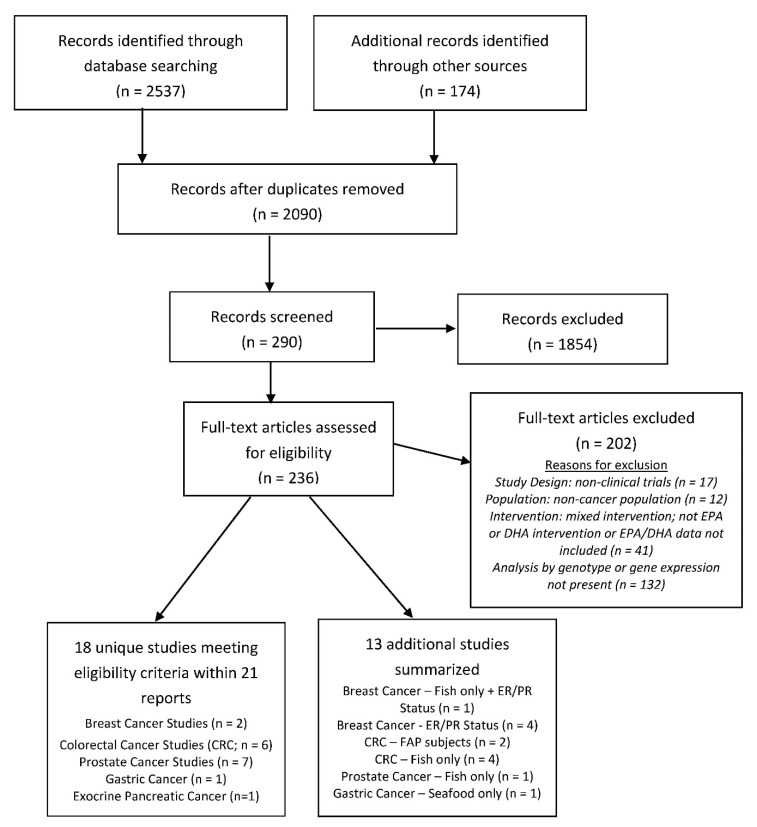
Flow diagram of the search and screen process used in study selection.

The initial database search yielded 1916 distinct citations. An additional 174 citations, identified via other sources, were also included for screening. Eighteen unique studies within 21 publications fit the eligibility criteria within our scoping review (Figure 2). Cancer types include breast cancer (*n* = 2), gastric cancer (*n* = 1), exocrine pancreatic cancer (*n* = 1), chronic lymphocytic leukemia (CLL; *n* = 1), prostate cancer (PCA; *n* = 7), and colorectal cancer (CRC; *n* = 6). Thirteen additional studies, as described above, were added to enhance the understanding of the research topic, i.e., fish intake only (*n* = 6); studies that involved subjects with FAP (*n* = 2); and studies that report on the estrogen receptor (ER) or progesterone receptor (PR) status of breast cancer tumors (*n* = 5) were summarized. 

**Figure 3:**
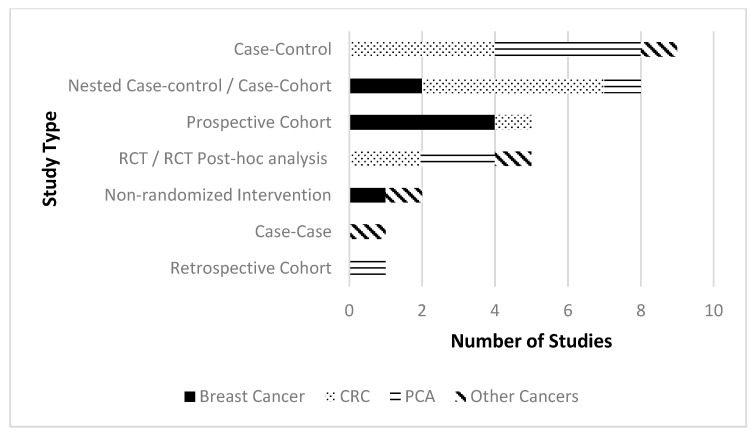
Number of studies by study design type split by cancer type.

Study characteristics and general results are described in Table 1. Most studies were observational, with case-control studies being the most common study design type (*n* = 9). Five studies were either randomized controlled trials (RCTs) or post-hoc analyses of RCTs. (See Figure 3) When possible, results of similar studies are synthesized as a group (according to cancer type and then study type). Details regarding genotypes, gene expression pathways and significant statistical results of individual studies can be found in Table 2.

### 3.2. Breast Cancer Studies

Two breast cancer studies within three reports met the inclusion criteria (see Table 2). A nested case-controlled study reported within two publications involving 399 breast cancer cases and 678 healthy controls reported no overall association between glutathione S-transferases (GST) genotypes and breast cancer risk. However, in post-menopausal women, differences in risk between high- and low-activity GST genotypes when stratifying risk by marine n-3 fatty acid intake was shown. Specifically, post-menopausal women with specific GST genotypes and consuming ≤ 200 mg marine n-3 fatty acids daily exhibited ~50% *decreased* cancer risk versus those consuming higher intakes of marine n-3 fatty acids [11]. In a kin paper, Ceschi et al. (2005), found that the heterozygous Cyclin D1 (CCND1) GA genotype *decreased* risk in all subjects compared to the GG genotype but the association was limited to women with high n-6 fatty acid intake or low marine n-3 intake or a total lack of certain GST genes. The effect was stronger in advanced disease [19]. 

Molfino et al. (2017) conducted an interventional, non-randomized, not placebo-controlled trial investigating DHA incorporation into red blood cells (RBC) after 2g DHA supplementation for 10 days in 11 healthy controls and 34 breast cancer patients. Statistically higher DHA incorporation in RBC membranes of BRCA 1 and 2 mutation carriers versus healthy controls was found [20]. Earlier, in vitro data in breast cancer cell lines demonstrated an increase in BRCA1 and BRCA2 mRNA expressions with DHA treatment, suggesting a transcriptional or post-transcriptional regulation of these genes by DHA [50]. Preclinical data also showed that DHA treatment increases BRCA1 protein level by 60% compared to an unsupplemented group and significantly reduced the incidence of breast cancer, potentially signifying a protective effect [51].

#### Additional Breast Cancer Studies—ER/PR Status

Results for an additional five studies that reported on ER/PR status are summarized (see Table 2). Four of the studies were prospective cohort studies involving 300,735 women resulting in 8402 breast cancer cases [21,23,24,25]. Two of these studies found a positive inverse association between n-3 LC-PUFA or fish intake, ER+/PR status and decreased breast cancer risk [23,24,25]. Stripp et al. (2003) found high total fish intake *increased* the risk of ER+ breast cancer [21], although individual fatty acid intakes were not measured.

Bassett et al. (2016) conducted a case-cohort study involving 571 breast cancer cases and 2492 healthy controls. High dietary intake of both EPA and DHA was also associated with *decreased* risk of ER+ breast cancer [22].

### 3.3. Colorectal Cancer Studies

Six studies within seven reports involving CRC fit the inclusion criteria as shown in Table 2. There were four case-controlled or nested case-controlled studies involving 5905 CRC cases and 5311 healthy controls [26,27,30,31]. A study by Habermann et al. (2013) found that carriers of prostaglandin-endoperoxide synthase 1 (PTGS1) rs10306110 (−1053 A > G) with low DHA intake resulted in an *increased* risk of colon cancer [26]. Another study by Theodoratou et al. (2008) found that subjects with wild-type and heterozygous APC 1822 and low intake levels of n-3 LC-PUFA had an *increased* CRC risk, while subjects with homozygous APC 1822 (TT) and low levels of n-3 LC-PUFA intake had a *decreased* CRC risk [31]. Stern et al. (2003) found that subjects with the poly ADP ribose polymerase (PARP) gene (rs1136410) and high marine n-3 LC-PUFA intake had an *increased* CRC risk. Further analysis revealed that this PARP gene modified associations between marine n-3 LC-PUFA and CRC risk only amongst rectal cases [30]. Kantor et al. (2004) found that both low and moderate genetic risk scores combined with increased dark fish or total EPA and DHA consumption resulted in a *decreased* CRC risk, while a high genetic risk score combined with an increased dark fish or total EPA and DHA consumption resulted in an *increased* CRC risk. When the genetic risk score was limited to six single-nucleotide polymorphisms (SNPs) associated with the transforming growth factor-beta (TGF-β) pathway, no interaction was found [27]. 

Song et al. (2015) conducted a prospective cohort study from which 1125 CRC cases were categorized as having microsatellite instability-high (MSI) tumors (a distinct phenotype of CRC) or microsatellite-stable (MSS) tumors. The authors found those with CpG island methylator phenotype (CIMP)-low/negative status and MSI tumors with increasing marine n-3 LC-PUFA intake (quartile 1 versus quartile 4) were associated with significantly *decreased* CRC risk [28]. 

A post-hoc analysis of an RCT investigated the expression of chemokine C-C motif ligand 2 (CCL2), a pro-inflammatory chemokine, in 55 CRC patients with liver metastases randomized to 2g EPA daily or placebo for an average of 30 days prior to surgery [32]. They found plasma CCL2 levels post-intervention *increased* in the placebo group and *decreased* in the EPA group. In the EPA group, 614 genes were identified as differentially expressed in tumor cells from patients with decreased CCL2 plasma levels compared to patients with no change or an increase in CCL2 plasma levels. Furthermore, EPA-treated patients whose plasma CCL2 levels decreased had a significantly better disease-free survival compared with individuals in whom the plasma CCL2 level increased after supplementation. A decrease in the synthesis of CCL2 and CCL2 receptor expression was also shown by Volpato with EPA treatment in vitro, suggesting a potential mechanism of action. Additionally, although EPA is known to inhibit PGE2 synthesis and EP4 receptor activation in CRC cells, Volpato’s in vitro data demonstrated EPA’s effects on CCL2 were PGE2-EP4 receptor-independent.

#### 3.3.1. Additional CRC Studies: Fish Intake Only 

An additional four case-cohort studies/case-controlled studies [33,34,35,36] involving in total 3506 CRC cases and 7618 heathy controls were identified (see Table 2). Three of the four studies did not find any significant interactions between fish intake, genotypes and CRC risk [34,35,36]. The final study by Anderson et al. (2013) found that IL10 rs3024505 homozygous wild-type carriers consuming 25g of fish per day had a 10 % *decreased* risk of CRC while variant carriers had no risk reduction with similar intake [33].

#### 3.3.2. Additional CRC Studies—FAP Patients 

Two studies involving participants with FAP were also analyzed (see Table 2). Almendingen et al. (2007) conducted a case-controlled study and found serum phospholipid DHA levels to be lower in healthy controls versus FAP patients [37]. An RCT by West et al. (2010) involved 58 FAP patients given either 2g EPA or placebo per day for six months. The EPA-FFA group experienced a *decrease* in the number of polyps, diameter of polyps and global polyp burden at six months versus the placebo group [38]. 

### 3.4. Prostate Cancer Studies 

Seven studies within eight reports involving PCA were identified (see Table 2). Four of the studies were case-controlled or nested case-controlled studies involving approximately 25460 PCA cases and 25800 healthy controls. These studies examined the interaction of various genotypes, fatty acid intake or levels and PCA risk and results were mostly positive [15,16,41,44]. Cheng et al. (2013) found that carriers of the myeloperoxidase (MPO) GA/AA genotype showed an *increased* risk of aggressive PCA with low EPA and DHA intake (quartile 1) versus carriers of the MPO GG genotype [41]. Fradet et al. (2009) found that cyclooxygenase-2 (COX-2) SNP rs4648310 AA carriers with an increasing intake of n-3 LC-PUFA was associated with a *decreased* risk of PCA. Furthermore, carriers of COX-2 rs4648310 (+8897 AG or GG) with low n-3 LC-PUFA intake were associated with an *increased* risk of PCA, and this was reversed by increasing n-3 LC-PUFA intake [15]. Hedelin et al. (2006) found that carriers of the COX-2 SNP (rs5275: 16365 T/C) who increased intake of salmon-type fish (once per week or more) versus those who never ate salmon-type fish had a *decreased* risk of PCA [16]. Khankari et al., (2016) with the use of a weighted PUFA-specific polygenic risk score (WPRS), found no overall association between the genetically predicted n-3 LC-PUFA evaluated and PCA risk. However, when stratified by age, modest *increases* in prostate cancer risk were observed for EPA among men > 62 years of age [44].

Cui et al. (2016) conducted a retrospective cohort investigating the association between fatty acid desaturase (FADS) rs174537 and Cg27386326 methylation status with n-3 LC-PUFA composition and markers of n-3 LC-PUFA biosynthesis in specimens from 60 PCA patients undergoing radical prostatectomy. When comparing FADS rs174537 GG versus TT genotypes, they found that lower DHA levels in the PCA tissue was associated with the TT genotype [42]. 

An RCT, involving 84 PCA patients, investigated how daily supplementation of 1098 mg EPA and 549 mg DHA or 30 mg lycopene or placebo for three months modulated several gene expression pathways. A canonical pathway analysis revealed statistically significant differences in the modulation of eight gene expression pathways in the fish oil group versus the placebo group at 3 months [39,40].

Galet et al. (2014) conducted a post-hoc analysis of an RCT that randomized 55 PCA patients to either a low-fat diet plus 1000 mg EPA and 1835 mg DHA or a Western diet for 4–6 weeks prior to a radical prostectomy. The low-fat diet supplemented with n-3 LC-PUFA resulted in a *decreased* prostate cancer tissue cell-cycle progression (CCP) score at post-intervention versus the Western diet group [43]. 

#### Addition Prostate Cancer Study: Fish Intake Only 

Catsburg et al. (2012) conducted a case-control study in which 497 localized and 936 advanced PCA cases and 760 controls were genotyped. Carriers of the C allele of prostaglandin-endoperoxide synthase 2 (PTGS2) 765 G/C with high white fish intake had an *increased* risk of advanced PCA risk, while an inverse association between dark fish intake and advanced PCA risk was found among carriers of the GG allele, but not among C allele carriers [45].

### 3.5. Studies Involving Other Cancers

Four studies involving other cancer types were also included in this review (see Table 2). Fahrmann et al. (2013) investigated Nuclear factor Kappa B (Nf-KB) activity levels in a non-randomized, uncontrolled study. Fifteen early stage chronic lymphocytic leukemia patients were supplemented with escalating n-3 LC-PUFA doses from 2.4 g to 7.2 g/day over 12 months with the highest n-3 LC-PUFA intake level associated with a *decrease* in Nf-KB activity in subjects with higher Nf-KB activity levels at baseline. There was also a significant *decrease* in mRNA abundance of 31 genes with higher Nf-KB activity at baseline in response to n-3 LC-PUFA intake [46].

In an RCT by Cury-Boaventura et al. (2012) involving 25 surgical gastric or colon cancer, a preoperative fish oil infusion (0.2 g/kg for 3 days) altered the expression of seven genes related to cell death demonstrating a protective effect on postoperative lymphocyte apoptosis while the medium/long-chain triglycerides infusion altered 12 genes with both pro-and antiapoptotic effects associated with postoperative lymphocyte and neutrophil apoptosis [47]. In a case-control study by Huang et al. (2014), *decreased* gastric cancer risk was associated with increased seafood intake (≥1 time/week) in carriers of toll-like receptor 4 (TLR4) gene CC/CT rs10116253 and TT/TC rs1927911 [49].

Morales et al. (2007) conducted a case-case study involving 121 pancreatic cancer patients and found that patients with K-ras-mutated tumors had a significantly lower intake of n-3 LC-PUFA compared to those with non-K-ras-mutated tumors [48].

## 4. Discussion

The current scoping review suggests that human research regarding the interaction between genetic variants, dietary n-3 LC-PUFA (foods, dietary supplements, or enteral/parental nutrition) or tissue status, and cancer risk and/or treatment is at an inaugural stage. Several databases devoted to the genomics of cancer demonstrates how much this research is in its infancy. The Catalogue of Somatic Mutations in Cancer, for example, houses data on over a million samples, including almost 3 million coding mutations and over 100 million abnormal expression variants [52]. In contrast, the studies analyzed in this review identified only a few hundred genotypes or gene expression pathways. Nonetheless, studies identified in the current review describe a broad range of complex interactions between functional genetic variants of cancer and n-3 LC-PUFA intake. Evidence in this review identified interactions between genetic variants of three main cancers: colorectal, prostate and breast and n-3 LC-PUFA intake. N-3 LC-PUFA exert anti-carcinogenic activity through a variety of proposed mechanisms, including decreased inflammation via the modulation of COX activity [53]. Increased risk or enhanced progression of various cancers has been attributed to COX-2 genetic variants [54,55]. The alteration of membrane dynamics and cell surface receptor function, and increased cellular oxidative stress (hypothesized to induced cancer cell apoptosis) are other anti-carcinogenic mechanisms linked to n-3 LC-PUFA [9,14,53]. The ability of n-3 LC-PUFA to regulate oncogene transcription factors has also been described, including, for example, decreased nuclear factor-kappa B (Nf-kB) activity making n-3 LC-PUFA potentially useful adjunctive cancer therapy agents by sensitizing tumor cells to chemotherapy and promoting apoptosis [56]. 

### 4.1. Breast Cancer Findings 

Two studies in the current review directly considered the interaction between genetic variants, breast cancer, and n-3 LC-PUFA intake. Consistent with the hypothesis that n-3 LC-PUFA may protect against cancer by promoting oxidative stress, the companion studies of Gago-Dominquez et al. (2004) [11] and Ceschi et al. (2005) [19] considered the role of GST variants in breast cancer. As glutathione-S-transferases reduce peroxidation products of oxidative stress, Gago-Dominquez et al. found that women possessing low activity GST genotypes had a lower risk of breast cancer if their diets were higher in n-3 LC-PUFA. In the same population, Ceschi and co-workers surprisingly found that the G870A variant of cyclin D1 (CCND1), which activates apoptosis in the presence of oxidative stress, reduced breast cancer risk when restricted to women with low n-3 LC-PUFA intake and high n-6 LC-PUFA intake (n-6 LC-PUFA also increases oxidative stress). High n-3 LC-PUFA intake was associated with lower risk but was not statistically significant, and levels of individual fatty acids were not measured; thus, these results should be considered preliminary. 

Another identified study considered the interaction between the BRCA gene mutation or tumor hormone status and n-3 LC-PUFA or fish intake [20] and showed that those with BRCA1/2 had higher RBC DHA levels. The BRCA gene mutation, like the APC mutation in CRC, appears to strongly predict genetic risk. While only 5%–10% of breast cancers are inherited, carriers of the BRCA gene mutations account for 45%–65% of heritable breast cancer [57]. Increases in BRCA mRNA expression and protein levels in human and cell studies have been reported in response to n-3 LC-PUFA supplementation, suggesting that n-3 LC-PUFA could be an important factor in reducing breast cancer risk [50,51]. 

As BRCA status is commonly related to tumor ER status [57], we examined four studies that considered the role of dietary [22,23,24,25] or tissue [22] n-3 LC-PUFA and ER status, and two that considered the role of fish intake and tumor hormone status [21,24]. Overall, the association between n-3 LC-PUFA intake (and/or fish intake) and breast cancer risk is consistent with those reported by Zheng et al.’s meta-analysis [58] and found that higher dietary n-3 LC-PUFA was associated with a lower risk of breast cancer, especially in ER+/PR patients, although no association was observed for fish intake alone. 

Ongoing trials currently registered in Clinicaltrials.gov appear to largely focus on the use of n-3 LC-PUFA as adjunctive treatment for breast cancer (e.g., NCT02831582; NCT02996240; NCT02278965; NCT01821833; NCT01478477) and prevention related to tumor hormone status (e.g., NCT02295059). Some of these trials may provide additional insights regarding genetic variants and dietary n-3 LC-PUFA.

### 4.2. Colorectal Cancer Findings

While a growing body of mechanistic evidence suggests that EPA and DHA play a protective role in CRC risk [56], evidence from human observational and clinical trials in the current review is complex and mixed. Of six studies examining an interaction between dietary DHA/EPA and gene expression or specific genetic variants in CRC risk, three found evidence suggestive of *reduced* risk for CRC [26,28,32], while three reported mixed results [27,30,31]. 

Studies reporting protective effects of dietary DHA/EPA [26,28,32] examined pathways associated with cellular inflammation. Consistent with dietary n-3 LC-PUFA as adjunctive therapy, Volpato et al. (2016) found decreased plasma CCL2, a pro-inflammatory chemokine with known roles in metastasis, and longer disease-free survival in response to supplemental EPA, in CRC subjects undergoing surgery for liver metastasis [32]. Song et al. (2015) and Habermann et al. (2013) considered pathways of inflammation activated by COX activity, known to be modulated by n-3 LC-PUFA. Song found that EPA/DHA intake was associated with a lower risk of micro-satellite instable (MSI) tumors through a proposed reduction in prostaglandin (PG) E2 pathways responsible for the loss of DNA mismatch repair activity [26,28]. Habermann et al. specifically investigated the role of dietary n-3 LC-PUFA in subjects with genetic variants known to increase PG-derived inflammatory markers (i.e., PTGS1 and PTGS2, genes of PG synthesis enzymes), and found inverse associations between CRC risk and increased intake of EPA or DHA. Consistent with an anti-inflammatory mechanism, Andersen et al. (2013) found homozygous wild-type carriers of the anti-inflammatory cytokine IL10 rs3024505 to have a significantly *decreased* CRC risk per increase in daily fish consumption [33]. 

Looking at the tumor suppressor APC gene, Theodoratou and co-workers found that low n-3 LC-PUFA intake was associated with an *increased* CRC risk for the wild-type and heterozygous APC 1822, but with a *decreased* CRC risk in those homozygous for the variant allele. This study among others, points to the complexity of genotypic nutrient interactions, and highlights the need for more intervention trials to include genotyping in evaluating therapeutic responses. APC gene defects are responsible for FAP which increases CRC risk [31]. Studies of EPA/DHA intake and status in individuals with FAP are limited. Findings from West et al. (2010) suggest that EPA supplements should be considered for chemoprevention in patients with FAP [38]. In contrast, Almendingen et al. (2006) suggest that FAP patients may have disordered fatty acid metabolism compared to healthy subjects and, as such, their optimal intakes may be different [37]. 

Mixed results of genotype interactions between dietary n-3 LC-PUFA versus fish consumption, may help explain the inconsistent findings of the systematic reviews and meta-analyses of CRC risk. Chen and co-workers (2015) found a non-statistically significant *decreased* risk of proximal colon cancer and a statistically significant *increased* risk of distal colon cancer with increased n-3 LC-PUFA intake in their meta-analysis, but did not examine genetic variants [59]. In contrast, Yu et al. (2014) reported a significantly *reduced* risk of CRC among higher fish consumers, although genetic variants were also not included. [60]. Most recently, the World Cancer Research Fund (WCRF) concluded that, although there is mechanistic evidence that dietary n-3 LC-PUFA reduce CRC risk, likely through influencing inflammatory pathways, there is limited data for a link between fish consumption and CRC risk, making the evidence “limited” but “generally consistent” for a protective effect of fish against CRC risk [61]. 

### 4.3. Prostate Cancer Findings

Of three studies finding protective benefits of n-3 LC-PUFA in Prostate cancer, Cheng and co-workers found evidence supporting increased oxidative stress as the mechanism responsible. Specifically, they examined the interaction between MPO variants and serum levels of n-3 LC-PUFA [41]. Those with the MPO GG variation were expected to have the greater ability to upregulate oxidative stress while those with GA/AA variants were compromised, i.e., with a 2-fold *increase* in aggressive prostate cancer risk among men with low n-3 LC-PUFA. Due to the unsaturated nature of n-3 LC-PUFA, these fatty acids are highly peroxidizable and capable of generating reactive oxygen species (ROS), which alters cellular redox states. Many tumor cells exhibit altered ROS pathways, which enable higher intracellular ROS levels in response to dietary n-3 LC-PUFA and may induce tumor cell apoptosis [53]. 

The remainder of studies reporting a protective benefit of fish or n-3 LC-PUFA intake investigated genetic variants associated with inflammatory pathways, again including PTGS2 and COX-2. Catsburg and co-workers (2012) found PTGS2 765 G/C genetic variants with high white fish consumption were positively associated with risk only among carriers of the C allele [45]. While those with the PTGS2 765 G/G variant (lower inflammatory activity) exhibited a *decreased* risk of advanced prostate cancer with higher dark fish (higher in n-3 LC-PUFA) consumption. The authors hypothesize that the C allele may be responsible for increased PTGS2 activity (hence greater inflammation) and that interactions with heterocyclic amines may have further increased inflammatory pathways and prostate cancer risk. 

Looking at variants of COX-2, Hedelin et al. (2006) found high intake of salmon-type fish but not n-3 LC-PUFA per se, among subjects who were heterozygous or homozygous for allele (C) of the SNP (rs5275: +6365 T/C) *decreased* prostate cancer risk by 72% [16]. It should be noted that, for many studies using a food frequency questionnaire (FFQ), intakes of each fatty acid are estimates based on subject recall, whereas blood levels of n-3 LC-PUFA are more accurate determinants of intake and tissue status [62].

Similarly, Fradet and co-workers found men with the COX-2 variant rs4648310 (+8897 A/G) to be at *increased* risk for prostate cancer when intake of n-3 LC-PUFA was low, but higher n-3 LC-PUFA intake was strongly protective [15]. SNP rs4648310 and rs5275 are located 2.4 kb apart and exhibit weak linkage disequilibrium in their populations, which suggests that either of these genetic variants may have effects on COX-2 activity. Evidence collectively suggests that n-3 LC-PUFA may modify prostate inflammation through the COX-2 pathway. Despite these observations, short-term supplementation of high-dose fish oil failed to reduce COX-2 gene expression in a small RCT conducted by Chan et al. (2011) [39], although they did not report the genetic variant distribution of their population. 

The results of three ongoing intervention trials (in Clinicaltrials.gov) promise to provide additional insights into the role on dietary n-3 LC-PUFA and prostate cancer risk and treatment (NCT02333435), with at least two of these being likely to provide evidence regarding the interaction of n-3 LC-PUFA with genetic variants (NCT03290417; NCT02176902). 

### 4.4. Findings in Other Cancer Types 

Studies involving patients with CLL [46], gastric [47,49], and pancreatic [48] cancers were also included in the current review and found specific polymorphisms of genes involved in inflammation pathways (e.g., TLR4) and cell proliferation or apoptosis (e.g., NF-kB, TRAF3, CSF1/2, K-ras oncogene) positively associated with higher intake of n-3 LC-PUFA and decreased cancer risk. Studies in these other cancer types were very limited and suggest that more clinical research is needed to elucidate potential benefits of n-3 LC-PUFA in these specific genetic variants of cancer.

### 4.5. Strengths and Limitations

In this scoping review, we have endeavored to provide a comprehensive summary of key study design elements on the topic of n-3 LC-PUFA and cancer that assist the reader in judging various aspects of evidence quality and strength. Scoping reviews are not intended to provide a systematic assessment of the quality or strength of evidence in a particular area [63,64], so the current review lacks a formal evaluation of risk of bias or evidence strength. It is not uncommon that, given the intent behind a scoping review to capture the state of the science on a broad topic, the study selection process can include post hoc, or modified, inclusion and exclusion criteria as new ideas emerge during the process of gathering and reviewing information [64]. Accordingly, during our study selection process, we modified our criteria to include studies that were found during screening (i.e., FAP patients and studies of fish for CRC; hormone receptor status for breast cancer) that enhanced the understanding of the research topic and strengthened our scoping review. 

Due to the nature of examining a broad research question on n-3 LC-PUFA and cancer, our review identified three main cancers, yet culminated in a heterogeneous collection of studies with various polymorphisms that precludes a simple summary, but does identify varied data and mechanisms of action as well as limitations of the existing evidence base. For example, Hou et al. (2016) suggest that the discrepancy between findings for CRC risk and fish consumption versus n-3 LC-PUFA intake may be due to the source of n-3 LC-PUFA, e.g., from fish oil, purified EPA, DHA, or a combination of the two resulting in variable ratios of EPA and DHA, along with the administration of different doses [56]. This observation is consistent with the evidence limitations for n-3 LC-PUFA and other chronic disease. For example, the potential dose–response relationship between dietary n-3 LC-PUFA and chronic disease prevention has been debated for decades in the cardiovascular disease arena, where the evidence base is much larger [65]. Additional evidence from controlled trials where n-3 LC-PUFA dosing is more defined will help evolve the evidence base regarding n-3 LC-PUFA intake level and cancer risk. Further complicating the interpretation of the current data is the use of predominately self-reported dietary intake methods [53], i.e., food frequency questionnaires, rather than biomarker data such as n-3 LC-PUFA blood/tissue levels. Food frequency questionnaires are not designed to provide an accurate estimate of absolute intake and have been criticized as a source of reporting/measurement error [66,67]. Finally, the current evidence base regarding associations between n-3 LC-PUFA intake or tissue levels and genotype-specific cancer risk is predominately observational. Chance, bias, and confounding must all be considered when interpreting results from a largely observational evidence base [68]. Randomized, controlled trials using identified genetic variants of cancer phenotypes and targeted n-3 LC-PUFA doses are needed to further assess potential anti-neoplastic response. It is hoped that insights gained from our scoping review will help further this field of investigation. 

## 5. Conclusions

Evidence from this scoping review suggests that dietary n-3 LC-PUFA may interact with genetic variants of inflammatory signals, apoptotic gene expression markers, and cell cycle regulation factors in a manner that may decrease the risk of breast, colorectal and prostate cancer. However, depending upon the genetic polymorphism identified, a few studies found an increased risk or no benefit. Results reported in the current review require replication in large cohorts and well-powered intervention trials with genotyping to further elucidate the role of dietary n-3 LC-PUFA and genetic variation in cancer risk. Common mechanisms for n-3 LC-PUFA’ anti-neoplastic effects include the inhibition of inflammatory pathways, e.g. COX-2 activity, PTGS, CCL2, and enhanced oxidative stress pathways and apoptosis, e.g., myloperoxidase genes and NF-kB. This review highlights variability in individual responses to n-3 LC-PUFA supplementation, some of which appear to be dose dependent. Once a genetic variant is identified, a targeted, personalized therapeutic approach that includes DHA and/or EPA may be possible as an adjuvant to immunotherapy or chemotherapy. The expansion of research designed to evaluate n-3 LC-PUFA interactions with genetic variants of other leading cancers, such as gastric, pancreatic, lung and bronchus, is also needed. 

## Figures and Tables

**Table 1:** Summary characteristics of 31 unique human studies reporting the relationship between risk and other cancer-related outcomes associated with dietary omega-3 long-chain polyunsaturated fatty acids (n-3 LC-PUFA) and genotype.

Characteristics	Breast (*n* = 7)	CRC (*n* = 12)	Prostate (*n* = 8)	Other (*n* = 4) ^1^
Study Type	Intervention ^2^ (*n* = 1), Prospective cohort (*n* = 4), nested case-control/case-cohort (*n* = 2)	RCT (*n* = 2) ^3^, Prospective cohort (*n* = 1), nested case-control/case-cohort (*n* = 5), case-control (*n* = 4)	RCT (*n* = 3), Retrospective cohort (*n* = 1), nested case-control/case-cohort (*n* = 1), case-control (*n* = 3)	Intervention (*n* = 1), RCT (*n* = 1), case-control (*n* = 1), case-case (*n* = 1)
Year Published (range)	2003–2017	2002–2016	2007–2016	2007–2014
Geographic Location	Australia (*n* = 1), China (*n* = 1), EU (*n* = 2), Japan (*n* = 1), USA (*n* = 2)	EU (*n* = 4), Singapore (*n* = 1), UK (*n* = 3), USA (*n* = 4)	Mixed (*n* = 1) ^4^, Sweden (*n* = 1), USA (*n* = 6)	Brazil (*n* = 1), China (*n* = 1), Spain (*n* = 1), USA (*n* = 1)
Total Participants ^5^	11,864	19,629	53,268	663
Average participants per trial (range)	1695 (43–3885)	1636 (26–2948)	1073 * (21–1433)	165 (20–511)
Baseline age range (years)	27–80	16–79	35–79	18–87
Gender distribution	100% female	42–49% female 50–57% male	100% male	28–53% female 47–72% male
Range of Intervention duration	10 days ^6^	12 days–6 months	4 weeks–3 months	3 days–12 months
Duration of follow-up	4.8–20 years	1–26 years	NA	NA
Mean DHA ± SD, mg/day (range)	1070 ± 1315 (140–2000)	200 ± 131 (85–341)	844 ± 882 (147–1835)	1800 ± 900 (900–2700)
Mean EPA ± SD, mg/day (range)	80 ^7^	624 ± 942 (15–2000)	733 ± 566 (72–1098)	2400 ± 1200 (1200–3600)
EPA+DHA, %kcal	0.19 ^7^	NR	NR	NR

^1^ Other includes—gastric, pancreatic, and chronic lymphocytic leukemia; ^2^ Intervention = non-randomized and/or not placebo controlled; ^3^ RCT=Randomized controlled trial may also include RCT post-hoc analysis; ^4^ Mixed consortium = EU, Australia, Malaysia, China, Japan, India, Africa, Canada and USA; ^5^ Total participants = completed subjects and cases/controls at longest duration of follow-up; ^6^ only one intervention trial, and, therefore, no range was given; ^7^ insufficient data to calculate the mean, SD, and range. NA = not applicable NR = data not reported; SD=standard deviation. * Average and range exclude the largest study of *n* = 45,755 subjects.

**Table 2:** Evidence table of included studies.

Citation	Study Type	Population Description *(N Entered/Completed)*	Intervention	Exposure Assessment	Genotypes/Gene Expression Assessed	Outcomes Assessed	Statistically Significant Results Related to Genotype/Gene Expression and n-3 LC-PUFA Intake/Levels	Interpretation of Results
***Included Breast Cancer Studies (n = 2 of 3 Reports)***								
Ceschi 2005 [19]; Gago-Dominguez 2004 [11]	Nested case-control	Chinese women: BCa patients (399/258) Healthy controls (678/670)	NA	*FFQ:* marine n-3 & n-6 LC-PUFA intake (quartiles)	CCND1 G870A GSTM1 null-null GSTMI positive GSTT1 null-null GSTT1 positive GSTP1 AA GSTPI AB/BB	BCa risk	***Ceschi 2005 Results*** **CCND1 GA vs. GG genotype:** low marine n-3 intake assoc. with decreased risk of BCa (OR = 0.54, 95% CI =0.32–0.93). *High marine n-3 intake (OR = 0.78*, 95% CI = 0.44–1.41) * CCND1 GA vs. GG genotype with advanced stage BCa: low marine n-3 intake assoc. with decreased risk of BCa (OR = 0.45, 95% CI =0.24–0.86). High marine n-3 intake (OR = 0.57 95% CI 0.26–1.26) * CCND1 GA vs. GG genotype: low marine n-3 and high n-6 intake assoc. with decreased risk of BCa (OR = 0.33, 95% CI = 0.15–0.73). High marine n-3 and high n-6 intake (OR = 0.69 95% CI 0.32–1.49) * CCND1 GA vs. GG genotype carrying GSTM1-null: low marine n-3 intake assoc. with decreased risk of BCa (OR = 0.19, 95% CI = 0.08–0.43) ** CCND1 GA vs. GG genotype carrying GSTT1-null: low marine n-3 intake assoc. with decreased risk of BCa (OR = 0.29, 95% CI = 0.11–0.73) ** Notes: Menopausal status did not modify assoc. btn CCND1 status and BCa risk. ***Gago-Dominguez 2004 Results*** *Post-menopausal women:* **GSTT1 null**: high (Q2–Q4) vs. low (Q1) marine n-3 intake assoc. with decreased risk of BCa (OR = 0.54, 95% CI = 0.29–1.00) (Note: borderline statistically significant) **GSTP1 AB/BB**: high (Q2–Q4) vs. low (Q1) marine n-3 intake assoc. with decreased risk of BCa (OR = 0.49, 95% CI = 0.26–0.93) **GSTM1 null-null and GSTP1 AB/BB**: high (Q2–Q4) vs. low (Q1) marine n-3 intake assoc. with decreased risk of BCa (OR = 0.36, 95% CI = 0.14–0.94) **GSTT1 null and GSTP1 AB/BB**: high (Q2–Q4) vs. low (Q1) marine n-3 intake assoc. with decreased risk of BCa (OR = 0.26, 95% CI = 0.08–0.78) *Pre-menopausal women:* No evidence of risk modification by genotype or gene-diet interaction.	*Ceschi 2005:* Positive (POS) *Gago-Dominguez 2004:* POS
Molfino 2017 [20]	Intervention trial, non-randomized, not placebo controlled	BRCA1/2 (12/11) Familial history of BCa (12/12) Sporadic BCa (10/10) Healthy Controls (11/10)	DHA 2g/day for 10 days	*RBC:* EPA, DHA, omega-3 Index *FFQ:* seafood intake	BRCA1 BRCA2	*Timepoints:* Baseline, day 10 DHA bio-availability	**BRCA1 or BRCA 2 gene mutation** predicted higher DHA levels in RBC membranes vs. healthy controls post-supplementation (β coefficient = 0.30; 95% CI = 0.05–0.55; *p* = 0.02) BCa type and self-reported seafood consumption: NS	Not Applicable (NA)
***Breast Cancer Studies—Fish Only + ER/PR Status (n = 1)***								
Stripp 2003 [21]	Prospective cohort study	23,693 post-menopausal women resulting in 424 cases (303 ER+ cases; 91 ER− cases; 30 cases unknown)	N/A	*FFQ:* total fish/lean fish/fatty fish intake; (g/day; quartiles)	ER status	*Follow-up:* median length of 4.8 years BCa risk	**ER+ BCa**: high total fish intake assoc. with increased rate of ER+ BCa (adjusted IRR per additional 25 g of mean daily intake of fish: 1.14; 95% CI = 1.03–1.26) **ER− BCa**: NS interaction	ER+: Negative (NEG) ER−: Not significant (NS)
***Breast Cancer Studies—ER/PR Status (n = 4)***								
Bassett 2016 [22]	Case-cohort	BCa cases (571/470) healthy controls (2492/2021)	NA	*%PPL fatty acids*: DHA, EPA, total n-3 LC-PUFA (quintiles) *FFQ:* DHA, EPA (g/day; quintiles)	ER/PR status	BCa risk *in relation to PPL and fatty acid intake stratified by ER+ or PR+ status*	**Total BCa incidence**: high EPA intake assoc. with decrease risk (HR = 0.83; 95% CI = 0.74–0.03; *p* = 0.001); high DHA intake assoc. with decreased risk (HR = 0.83; 95% CI = 0.74–0.94; *p* = 0.002) **ER+/PR+ BCa**: high EPA dietary intake (Q5 vs. Q1) assoc. with decreased risk of ER+ BCa (HR = 0.82; 95% CI = 0.72–0.94; *p*=0.004); high DHA dietary intake (Q5 vs. Q1) assoc. with decreased risk of ER+ BCa (HR = 0.84; 95% CI = 0.73–0.96; *p* = 0.01) **ER−/PR− BCa**: NS interaction	ER+: POS ER−: NS
Kim 2006 [23]	Prospective Cohort	121701 (80375 completed) postmenopausal women resulting in 3537 cases	N/A	*FFQ*: n-3 LC-PUFA (% of energy)	ER/PR status	*Follow-up:* 20 years BCa risk	**ER/PR status** and n-3 LC-PUFA intake: NS interaction	NULL
Kiyabu 2015 [24]	Prospective Cohort	55541 (38234 completed) Japanese women aged 45–74 resulting in 556 cases of BCa	N/A	*FFQ:* total n-3, DHA, EPA, fish, n-3 LC-PUFA-rich fish intake (g/day; quartiles)	ER/PR status	*Follow-up:* 14.1 years BCa risk	**ER+ PR+ BCa** and fish or n-3 LC-PUFA-rich fish intake: NS **ER+/PR+ BCa**: increasing EPA intake assoc. with decreased risk across quartiles of intake and statistically significant decreased risk btn Q2 vs. Q1 (multivariable-adjusted HR Q2 vs. Q1 = 0.47; 95% CI = 0.25–0.89; *p* trend 0.47). *Note: EPA was overall protective but only statistically significant at Q2.*	POS
Park 2011 [25]	Prospective Cohort	99800 (85089 completed) post-menopausal women resulting in 3885 cases of invasive BCa	N/A	*FFQ*: EPA, DHA (g/1000 kcal; quintiles)	ER/PR status	*Follow-up:* 12.4 years BCa risk	**ER/PR status** and EPA or DHA intake: NS **ER+/PR− BCa**: increasing EPA intake assoc. with decreased risk across quartiles of intake and statistically significant decreased risk btn Q4 vs. Q1 (multivariable-adjusted HR Q4 vs. Q1 = 0.70; 95% CI = 0.50–0.99; *p* trend 0.18). *Note: EPA was overall protective but only statistically significant at Q2.*	POS
***Included Colorectal Cancer Studies (n = 6 of 7 Reports)***								
Habermann 2013 [26]	Case-control	Colon cancer patients (1574/1543) Rectal cancer patients (791/712) Healthy controls to colon cancer cases (1970/1900) and rectal cancer cases (999/912)	NA	*FFQ (CARDIA diet history question-naire):* EPA, DHA and total PUFA and total n-3 LC-PUFA intake (tertiles)	107 candidate polymorphisms and tagSNPs within: PTGS1 PTGS2 ALOX12 ALOX5 ALOX15 FLAP	CRC risk	**PTGS1 rs10306110 (−1053 A > G) variant genotypes (AG/GG)**: low DHA intake assoc. with increased CRC risk (OR = 1.62, 95% CI = 1.14–2.30, adj. *p*-interaction 0.003, Bonferroni adj.- *p* = 0.06); low EPA intake assoc. with increased CRC risk (OR = 1.56, 95% CI = 1.09–2.22, *p*-interaction 0.006, Bonferroni adj. *p* = 0.10) **ALOX15 rs11568131 (10,339 C > T) wild type (GG genotype)**: high EPA intake assoc. with decreased CRC risk (OR = 0.80, 95% CI = 0.65–0.98, p-interaction 0.02; Bonferroni adj *p* = 0.36.)	POS
Kantor 2014 [27]	Nested case-control	CRC patients (260/260) Healthy controls (250/250)	NA	*FFQ:* Average 10-year fish oil supplement use (none, low, high); dark fish intake, dietary and total EPA and DHA (quartiles)	rs6691170(1q41) rs6687758(1q41 rs10936599(3q26.2) rs16892766(8q23.3) rs6983267(8q24.21) rs10795668(10p14) rs3802842(11q23.1) rs11169552(12q13.13) rs7136702(12q13.13) rs4444235(14q22.2) rs4779584(15q13.3) rs9929218(16q22.1) rs4939827(18q21.1) rs10411210(19q13.1) rs961253(20p12.3) rs4925386(20q13.33) *Note:* A genetic risk score was created by a tally of risk alleles present in the above 16 SNPs located within identified CRC susceptibility loci.	CRC risk	**Overall genetic risk** and dark fish intake: significant interaction (*p* = 0.009) **Overall genetic risk** and EPA + DHA intake: significant interaction (*p* = 0.02) **Lowest tertile of genetic risk**: increasing dark fish intake assoc. with decreased CRC risk (Q4 OR = 0.13, 95% CI = 0.04–0.48); increasing total EPA + DHA intake assoc. with decreased CRC risk (Q4 OR = 0.23, 95% CI = 0.07–0.78) **Mid tertile of genetic risk**: increasing dark fish intake assoc. with decreased CRC risk (Q4 OR = 0.14 95% CI = 0.04–0.53); with increasing total EPA + DHA intake assoc. with decreased CRC risk (Q4 OR = 0.43 95% CI = 0.21–1.41). **Highest tertile of genetic risk**: increasing dark fish intake assoc. with increased CRC risk (Q4 OR = 1.59; 95% CI = 0.51–4.97); with increasing total EPA + DHA intake assoc. with increased CRC risk (Q4 OR = 5.79 95% CI = 1.79–18.7) When the genetic risk score was limited to 6 SNPs assoc. with the TGF-β pathway, no interaction was observed (results not shown)	*Low and mid tertiles of genetic risk:* POS *Highest tertile of genetic risk:* NEG
Song 2015 [28]; Song 2016 [29]	Prospective cohort	*Song 2015* Participants (173,230/NR) resulting in 2501 CRC cases of which 1125 specimens were observed for MSI status *Song 2016* Participants (173,229/125,172) resulting in 2504 CRC cases of which 614 specimens were observed for T-cell infiltration in the tumor micro-environment	None	*FFQ*: marine n-3 LC-PUFA intake (quartiles, stratified by gender); fish oil supplement use, fish, EPA and DHA intake (stratified by marine n-3 LC-PUFA quartiles and gender)	CIMP: CACNA1G, CDKN2A (p16), CRABP1, IGF2, MLH1, NEUROG1, RUNX3, SOCS BRAF (codon 600): Wild-type, Mutated T-cells classified as either high or low-level infiltrate in CRC tumor tissue: CD3+, CD8+, CD45RO (PTPRC)+, or FOXP3+	*Follow-up: * 24–26 years MSI status CRC risk	***Song 2015 Results*** **CIMP-low/negative status, high microsatellite instability CRC tumor**: increasing marine n-3 LC-PUFA intake assoc. with decreased CRC risk (Q1 vs. Q4 intake HR 0.28; 95% CI = 0.12–0.66; *p*-trend = 0.02) ***Song 2016 Results*** **FOXP3+ T-cell high CRC tumors**: higher marine n-3 LC-PUFA intake assoc. with decreased CRC risk (*p* for heterogeneity = 0.006; regardless of MSI status) **FOXP3+ T-cell high CRC tumors**: higher marine n-3 LC-PUFA intake (≥0.35 g/day vs. <0.15 g/day) assoc. with decreased CRC risk (multivariable HR = 0.57, 95% CI = 0.40–0.81, *p* for trend < 0.001.) **FOXP3+ T-cell high CRC tumors**: higher EPA intake (Q3 and Q4 vs. Q1) assoc. with multivariable HR = 0.67, 95% CI = 0.49–0.93 and multivariable HR = 0.61, 95% CI = 0.44–0.86 respectively, p for trend = 0.003.0.86 respectively, *p* for trend = 0.003. **FOXP3+ T-cell high CRC tumors**: higher DHA intake (Q3 and Q4 vs. Q1) assoc. with multivariable HR = 0.58, 95% CI = 0.41–0.80 and multivariable HR = 0.65, 95% CI = 0.46–0.90 respectively, *p* for trend = 0.0010.90 respectively, *p* for trend = 0.001. **CD3+, CD8+, or CD45RO+ cell densities**: NS association	POS
Stern 2009 [30]	Nested case-control	Colon cancer patients (180/180 Rectal cancer patients (131/130) Healthy controls (1181/1176)	None	*FFQ:* total n-3 LC-PUFA and marine n-3 intake (low and high intake)	XRCC1: Arg194Trp (rs1799782), Arg399Gln (rs25487) OGG1: Ser326Cys (rs1052133) PARP: Val762Ala (rs1136410), Lys940Arg (rs3219145) XPD: Asp312Asn (rs1799793), Lys751Gln (rs13181)	CRC risk	**PARP gene (rs1136410)**: high marine n-3 intake assoc. with increased rectal cancer risk (OR = 1.7, 95% CI = 1.1–2.7, *p* = 0.016) Marine n-3 LC-PUFA and genotype interaction were NS for colon cancer.	*Rectal*: NEG *Colon:* NULL
Theodoratou 2008 [31]	Case-control	Patients with adeno-carcinoma of colorectum (2789/1656) Healthy controls (2749/2292)	None	*FFQ:* EPA, DHA and n-3 LC-PUFA intake (tertiles)	APC 1822 APC 1317	CRC risk	**Wild-type or Heterozygous APC 1822** (case-only): low EPA intake (OR = 0.45, 95% CI = 0.26–0.78; *p*-int = 0.02) or low DHA intake (OR = 0.43, 95% CI = 0.25–0.75; *p*-int = 0.01) assoc. with decreased CRC risk. **Wild-type or Heterozygous APC 1822**: low n-3 LC-PUFA intake assoc. with increased CRC risk **Variant (Homozygous) APC 1822**: low n-3 LC-PUFA intake assoc. with decreased CRC risk (*p*-int for case-only = 0.09, NS) **APC 1317 genotypes**: high dietary EPA or DHA intake assoc. with decreased risk but NS	MIXED RESULTS
Volpato 2016 [32]	RCT post-hoc analysis	Clinical samples from RCT of patients undergoing liver surgery for CRC liver metastasis (CRCLM) EPA 2g/day (29/29) Placebo (26/26)	EPA 2g/day for 12–65 days (average 30 days) Vs. Placebo	*FFQ*: n-3 LC-PUFA levels at baseline, before surgery and 6 weeks after discharge. *CRCLM tissue*: EPA levels	CCL2 plasma levels Genome-wide transcriptional profiling of tumors	*Timepoints:* pre-treatment, post-treatment CCL2 plasma and tissue levels	**Plasma CCL2 levels** with EPA intake vs. Placebo post-treatment: decreased CCL2 levels (*p* = 0.04) before liver resection. Reduction in plasma CCL2 following EPA treatment predicted improved disease-free survival (HR 0.32; 95% CI = 0.05–0.51, *p* = 0.003). Lack of ‘CCL2 response’ to EPA (i.e., increase or no change in CCL2 following treatment) was assoc. with a specific CRCLM gene expression signature. The authors concluded that reduction in plasma CCL2 in patients with CRCLM treated with EPA predicts better clinical outcome and a specific tumor gene expression profile.	POS
***Colorectal Cancer Studies—Fish Only (n = 4)***								
Andersen 2013 [33]	Case-cohort	CRC patients (970/970) Healthy controls (1897/1789)	N/A	*FFQ:* fish intake (g/day; tertiles)	IL10 C-592A(rs1800872) C-rs3024505-T IL1b C-3737T (rs4848306) G-1464C (rs1143623) T-31C (rs1143627) PTGS2 (encoding COX-2) A-1195G (rs689466) G-765C (rs20417) T8473C (rs5275)	CRC risk	**IL10 rs3024505 homozygous wild-type carriers (CC) vs. variant carrier (CT-TT)**: per 25 g fish/day assoc. with decreased CRC risk (CC adjusted IRR = 0.90; 95% CI—0.82–0.99) vs. CT-TT IRR = 1.08; 95% CI—0.94–1.24, *p*-interaction = 0.0065).	POS
Luchtenborg 2005 [34]	Case-cohort	CRC patients (929/588) Healthy controls (3346/2948)	N/A	*FFQ*: fish intake (g/day; quartiles)	APC mutation status hMLH1 expression	CRC risk	**APC gene mutation or hMLH1 expression** and fish intake levels: NS.	NULL
Slattery 2010 [35]	Case-control	Rectal cancer patients with primary tumor in the rectosigmoid junction or rectum (1505/750) Healthy controls (1838/1250)	None	*FFQ (CARDIA diet history questionnaire)*: total n-3 LC-PUFA and fish intake (tertiles)	TP53 gene mutations: codons 175, 245, 248, 237, 283 K-RAS2 mutations: codons 12 and 13 CIMP markers (phenotype): MINT1, MINT2, MINT31, CDKN2A (p16), MLH1	Risk of having genetic rectal tumor mutations	**CIMP+, TP53 or KRAS2 gene mutation tumors** and fish intake levels: NS *Note: Participants carrying CIMP with higher levels of n-3 LC-PUFA were assoc. with a twofold increased risk of a CIMP+ tumor however*, *type of n-3 LC-PUFA was not defined*	NULL
Tiemersma 2002 [36]	Nested case-control	CRC patients (NR/102) Healthy controls (NR/537)	N/A	*FFQ*:fish intake (servings/month; 0–1, 1–4, 4+ servings)	NAT1 and NAT2 GSTM1 genotype	CRC risk	**NAT1, NAT2 or GSTM1** with fish intake: NS	NULL
***Colorectal Cancer Studies—FAP Studies (n = 2)***								
Almendingen 2006 [37]	Case-control	FAP patients (38/NR) Healthy Controls (160/NR)	N/A	*FFQ (FAP patients) and 14-day diet diaries; (controls): * total n-3 LC-PUFA, DHA, EPA intake (% total energy) *Serum PPL:* total n-3 LC-PUFA, DHA, EPA	FAP	PPL in FAP patients vs. healthy controls	**DHA PPL levels (weight %)**: lower in controls vs. FAP patients (difference: −5.26, 95% CI = −6.25 to −4.28, *p* ≤ 0.0001) **DHA PPL levels (mg/L)** lower in controls vs. FAP patients (difference: −62.5, 95% CI = −78.14 to −46.83, *p* ≤ 0.0001) Mean dietary intake of DHA similar btn groups	NA
West 2010 [38]	RCT	Patients with FAP undergoing endoscopic surveillance of their retained rectum post-colectomy (58/55)	2g EPA-FFA/day vs. placebo for 6 months	fatty acid content of rectal mucosa	genes assoc. with FAP (APC)	Number and size of polypsGlobal rectal polyp burdenMucosal fatty acid content	**EPA-FFA vs. Placebo at 6 months**: Larger decrease in number of polyps: (difference btn groups −1.06; 95% CI = −1.78 to −0.35; *p* = 0.005) Larger % change in # of polyps: (difference btn groups −22.4%; 95% CI = −39.6 to −5.1; *p* = 0.012) Larger % decrease in polyp diameters: (difference btn groups −29.8%, 95% CI: −56.1 to −3.6%, *p* = 0.027). Global polyp burden: EPA-FFA group remained stable while placebo group worsened (difference btn groups 0.42, CI = 0.10–0.75, *p* = 0.011)	POS
***Included Prostate Cancer Studies (n = 7 of 8 Reports)***								
Chan 2011 [39]; Magbanua 2011 [40]	RCT	Men with low-burden PCA randomized to: Fish oil (27/21) Lycopene (29/22) Olive oil placebo (28/26)	EPA 1098mg + DHA 549mg/day for 3 months Vs. Lycopene 30mg/day for 3 months Vs. Olive oil placebo for 3 months	FFQ	***Chan 2011*** COX-2, IGF-I, IGF-IR gene expression in prostate tissue ***Magbanua 2011*** Gene expression pathways: Alanine and Aspartate Metabolism Aminoacyl-tRNA Biosynthesis Androgen and Estrogen Metabolism Apoptosis Signaling Arachidonic Acid Metabolism Axonal Guidance Signaling Biosynthesis of Steroids Butanoate Metabolism C21-Steroid Hormone Metabolism Caveolar-mediated Endocytosis CD27 Signaling in Lymphocytes Cell Cycle: G1/S Checkpoint Regulation Ceramide Signaling Cyanoamino Acid Metabolism DHA Signaling Endoplasmic Reticulum Stress Pathway GABA Receptor Signaling Galactose Metabolism Glutathione Metabolism Glycosaminoglycan Degradation Glycosphingolipid Biosynthesis–Ganglioseries Hepatic Cholestasis Inositol Metabolism Insulin Receptor Signaling LPS/IL-1 Mediated Inhibition of RXR Function Metabolism of Xenobiotics by Cytochrome P450 Methane Metabolism Methionine Metabolism N-Glycan Biosynthesis N-Glycan Degradation Nitrogen Metabolism Nrf2-mediated Oxidative Stress Response Oxidative Phosphorylation Pantothenate and CoA Biosynthesis PXR/RXR Activation Selenoamino Acid Metabolism Sonic Hedgehog Signaling Sphingolipid Metabolism Stilbene, Coumarine and Lignin Biosynthesis Tryptophan Metabolism Ubiquinone Biosynthesis	*Timepoints:* baseline and 3-month ***Chan 2011*** Changes in normal tissue gene expression biopsies in IGF-1 and in COX-2 ***Magbanua 2011*** Gene expression pathways modulated by interventions	***Chan 2011 Results*** Fish oil vs. placebo: NS change in IGF-1 and COX-2 gene expression in subjects ***Magbanua 2011 Results*** NS changes (after adjustment) in individual gene expression were detected in normal prostate tissue after fish oil supplementation. Canonical pathway analysis *, however, suggests statistically significant modulation of the following pathways in subjects taking **fish oil supplementation vs. placebo at 3 months**: Arachidonic acid metabolism (*p* = 0.0135) Nuclear factor (erythroid derived-2) factor 2 or Nrf2-mediated oxidative stress response (*p* = 0.0123) Glutathione metabolism (*p* = 0.0204) Cyanoamino Acid Metabolism (*p* = 0.0209) Metabolism of Xenobiotics by Cytochrome P450 (*p* = 0.0316) Alanine and Aspartate Metabolism (*p* = 0.0324) GABA Receptor Signaling (*p* = 0.0437) Nitrogen Metabolism (*p* = 0.0457) * For unadjusted, statistically significant changes in individual gene expression please consult original publication	*Chan 2011:* NULL *Magbanua 2011:* NA
Cheng 2013 [41]	Nested case-control	Current/former smokers with workplace asbestos exposure within last 15 years: PCA patients (724/641) Healthy controls (1474/1398)	NA	PPL fatty acid levels (quartiles)	MPO G-463A (rs2333227)	PCA risk	**MPO GA/AA vs. GG genotype**: low EPA + DHA (Q1) assoc. with increased risk of aggressive PCA (OR = 1.97, 95% CI = 1.07–3.63).	POS
Cui 2016 [42]	Retro-spective Cohort	Specimens from PCA patients undergoing radical prostatectomy (60/60)	NA	Prostate tissue fatty acids	FADS (rs 174537) (GG, GT, TT) Cg2736326 methylation status	Assoc. btn FADS rs174537, Cg27386326 methylation status with n-3 LC-PUFA composition, and markers of n-3 LC-PUFA biosynthesis.	**FADS rs 174537 GG vs. TT**: lower PCA tissue DHA levels assoc. with TT genotype (median 2.11 %total fatty acids, IQR = 1.79 = 3, mean difference = −0.75, *p* = 0.03)	NA
Fradet 2009 [15]	Case-control	Aggressive PCA patients (506/466) Healthy controls (506/478)	NA	*FFQ*: EPA, DHA, total n-3 LC-PUFA (quartiles); dark/white/fried fish, shellfish, tuna intake (tertiles)	COX-2: rs689466, rs20417, rs2745557, rs5277, rs2066826, rs5275, rs2206593, rs689470 and rs4648310	PCA risk	**COX-2 rs4648310 AA**: increasing n-3 LC-PUFA intake assoc. with decreased PCA risk (OR = 0.61, 95% CI = 0.47–0.81; *p* trend 0.006). **COX-2 rs4648310 (+8897 AG or GG)**: low n-3 LC-PUFA intake assoc. with increased PCA risk (OR = 5.49; 95% CI = 1.80–16.7) and reversed by increasing n-3 LC-PUFA intake (OR = 0.42; 95% CI = 0.13–1.37).	POS
Galet 2014 [43]	RCT post-hoc analysis	PCA patients 4–6 weeks prior to radical prostectomy randomized to: Low-fat diet (29/27) Western diet (26/21)	Low-fat diet plus EPA 1000mg + DHA 1835mg/day for 4–6 weeks vs. Western Diet for 4–6 weeks	*Serum/RBC*: EPA, DHA, total n-3	CCP score *Note: CCP is a validated genetic risk score for predicting recurrence after radical prostatectomy and death from PCA.* *The CCP score was calculated as average expression of 31 CCP genes*, *normalized to 15 housekeeper genes.*	*Timepoints:* pre-intervention, post-intervention Serum proin-flammatory eicosanoids LTB4 15(S)-HETE CCP score (genetic risk score)	**CPP score**: low-fat fish oil resulted in decreased PCA tissue CCP score post-intervention vs. western diet (*p* = 0.03).	POS
Hedelin 2006 [16]	Case-control	PCA patients (1499/1378) Healthy controls (1130/782)	NA	*FFQ*: n-3 LC-PUFA, EPA+DHA intake (quartiles); various types of fish intake (tertiles)	COX-2: rs2745557 (1202 C/T) rs20432 (13100 T/G) rs4648276 (13935 T/C) rs5275 (16365 T/C) rs689470 (18365 C/T)	PCA risk	**COX-2 gene (rs5275: 16365 T/C)**: salmon-type fish intake 1x/week or more vs. never assoc. with decreased PCA risk (OR = 0.28, 95% CI = 0.18–0.45; *p* trend < 0.01), **All genotypes** and intake of white fish, shellfish, herring/mackerel or EPA/DHA fatty acids: NS	*EPA/DHA:* NULL *Salmon intake*: POS
Khankari 2016 [44]	Case-control	PCA patients (NR/22721) Healthy controls (NR/23034) Total participants (48056/45755)	NA	Predicted plasma% of total fatty acids	*directly genotyped*: rs780094, rs2236212, rs174538 *imputed: * rs3734398, rs3798713, rs1074011, rs174547 rs2727270, rs1696695	PCA risk	When using the weighted PUFA-specific polygenic risk score (WPRS) no overall association was observed btn the genetically-predicted n-3 LC-PUFA evaluated and PCA risk. However, when stratified by age, modest increases in PCA risk were observed for EPA (OR = 1.04, 95% CI = 1.01–1.06) among men >62 years of age.	NEG
***Prostate Cancer Studies—Fish Only (n = 1)***								
Catsburg 2012 [45]	Case-control	Localized and advanced PCA patients (1800/1433) Healthy controls (1139/760)	N/A	*FFQ*: dark and white fish intake (never, low, high intake)	GSTP1: Ile105Val, rs1695 PTGS2: −765 G/C, rs20417 CYP1A2: −154 A/C, rs762551 EPHX1: Tyr113His, rs1051740 CYP1B1: Leu432Val, rs1056836 NAT2: Ile114Thr, rs1799930; Arg197Gln. rs1799931; Gly286Glu, rs1801279; Arg64Gln, rs180120 UGT1A6: Thr181Ala. rs110587; Arg184Ser, rs2070959 GSTM1: Null or present GSTT1: Null or present	PCA risk	**PTGS2 765 G/C**: high white fish intake vs. no/rare white fish intake assoc. with increased advanced PCA risk (adjusted OR = 1.85; 95% CI 1.19–2.89), stronger assoc. with well-done white fish (adjusted OR = 2.17 (1.05–4.48), NS after Bonferroni adjustment. **PTGS2 765 C/C**: high white fish intake vs. no/rare white fish intake assoc. with >3-fold increased advanced PCA risk (adjusted OR = 3.56; 95% CI = 1.61–7.88). **PTGS2 765 G/G**: high dark fish intake assoc. with decreased advanced PCA risk (adjusted OR = 0.53; 95% CI 0.35–0.80), NS after Bonferroni adjustment, data not shown.	*White Fish:* NEG *Dark Fish:* POS
***Included Other Cancer Studies (n = 3)***								
Fahrmann 2013 [46]	Uncon-trolled non-randomized interven-tion pilot study	Patients with CLL in the early stages (Rai Stage 0–1) (15/15) Healthy patients to establish normal values for NFκB activation in lymphocytes (no intervention given; *n* = 5)	Monthly escalating dosages up to 12 months: EPA 1200mg + DHA 900mg/day EPA 2400mg + DHA 1800mg/day EPA 3600mg + DHA 2700mg/day	Plasma fatty acids	NF-kB pathway Expression of 32 genes in lymphocytes *Note:* *NFκB may play a key role in controlling apoptosis and disease progression in* *hematologic malignancies with previous evidence showing inhibition of NFκB activation* in vitro *resulted in apoptosis of the malignant cells.*	*Timepoints:* baseline, 2.4g, 4,8g, 7.2g and 3 months post-intervention. Suppression of Nf-KB activity in lymphocytes in CLL patients. Doxorubicin sensitivity, mRNA lymphocyte expression	**NF-κB activity in patients with higher baseline NF-κB activation levels**: Decreased NFκB activity following 7.2g n-3 LC-PUFA/day vs. baseline (*p* = 0.027) Decreased NF-κB activity at 3 months post-intervention vs. baseline (*p* = 0.040) 7.2g n-3 LC-PUFA/day intake returned NFκB activity to levels comparable to control patients (mean ± SD: 87,138 ± 79,040 NFκB luminescence units/μg protein). **mRNA abundance of any genes in patients with higher baseline NF-kB activation levels**: significant decrease in abundance of 16 of the 31 identified mRNA genes vs. baseline **NF-κB activity in patients with lower baseline NF-kB activation levels**: NS. However, 7.2g n-3 LC-PUFA/day returned NF-κB activity to levels comparable to control patients. **mRNA abundance of any genes in patients with lower baseline NF-kB activation levels**: NS	POS
Cury-Boaventura 2012 [47]	RCT	Surgical patients with gastric or colon cancer (25/25)	Omegavenos^®^ (pure fish oil; 12/NR) Lipovenos^®^ (medium/long-chain triglycerides and soybean oil; 13/NR) Both given 0.2 g/kg body weight 10% for 3 days post-surgery	NR	800 genes related to inflammation, of which 108 were involved in cell death detected by 16-Assay Bioarray Hybridization and Detection	*Timepoints:* Baseline (t0), after infusion (t1), 3rd post-operative day (t2) leukocyte death, cell viability, apoptotic markers, and expression of genes assoc. with cell death	Up regulation expression of genes with fish oil emulsion: - 3 genes related to cell death: TNF receptor–assoc. factor 3 [TRAF3]; BCL2-assoc. athanogene 4 [BAG4]; non-metastatic cells 1 [NME1] protein [NM23A] - 2 genes related to cell proliferation: macrophage colony-stimulating factor 1 [CSF1]; granulocyte-macrophage colony-stimulating factor 2 [CSF2]) Down regulation expression of genes with fish oil emulsion: - 2 genes related to cell death: bifunctional apoptosis regulator [BFAR]; growth arrest and DNA damage–inducible alpha [GADD45A]	POS
Morales 2007 [48]	Case-case	Pancreatic cancer patients with K-ras mutation (94/83) without K-ras mutation (wild-type; 27/24)	None	*FFQ*: n-3 intake (tertiles); fish and shellfish (low, medium, high)	K-ras gene	Risk of K-ras mutation	**K-ras mutated vs. without K-ras mutation**: fish/seafood intake: NS; high n-3 fatty acids intake assoc. with decreased risk of K-ras mutation (OR = 0.19; 95% CI = 0.05–0.81; *p* = 0.024) *Note: type of n-3 PUFA undefined.*	POS
***Other Cancer Studies—Seafood Only (n = 1)***								
Huang 2014 [49]	Case-control	Gastric cancer patients (217/217) Healthy controls (294/294)	NA	*FFQ*: seafood intake (servings/week)	TLR4: rs10116253 (TT, TC, CC, TC/CC) rs1927911 (CC, CT, TT, CT/TT)	Gastric cancer risk	**TLR4 rs10116253 CC/CT genotype**: seafood intake assoc. with decreased risk gastric cancer (<1/week OR = 0.60, 95% CI 0.38–0.94; >1 time/week OR =0.09, 95% CI = 0.03–0.24) vs. TT (>1 time/week OR = 0.27, 95% CI = 0.11–0.65), NS interaction btn polymorphisms **TLR4 rs1927911 CT/TT genotype**: seafood intake (≥1 time/week) assoc. with reduced gastric cancer risk (OR = 0.09, 95% CI = 0.03–0.26) vs. CC (OR = 0.29, 95% CI = 0.12–0.71), NS interaction btn polymorphisms	POS

* High marine n-3 intake assoc. with decreased risk but NS ** When analysis was restricted to subjects with both low activity GST genotypes and low n-3 or high n-6 intake authors report “sparse data and unstable OR estimates” (sample sizes not reported). Abbreviations: APC = adenomatous polyposis coli (gene); ALOX# = arachidonate #-lipoxygenase; assoc. = associated; btn = between; BCa = breast cancer; BRAF = B-Raf proto-oncogene; BRCA = BReast CAncer susceptibility (gene); CCL2 = C-C motif ligand 2 (chemokine); CCND1 = Cyclin D1 (gene); CCP = cell-cycle progression; CI = confidence interval; CIMP = CpG island methylator phenotype; CLL = chronic lymphocytic leukemia; COX-2 = cyclooxygenase-2; CRC = colorectal cancer; CYP = cytochrome; DHA = docosahexaenoic acid; EPA = eicosapentaenoic acid; EPHX1 = epoxide hydrolase 1; ER = estrogen receptor; ER+ = estrogen receptor positive; ER− = estrogen receptor negative; FADS = fatty acid desaturase; FAP = familial adenomatous polyposis; FFQ = food frequency questionnaire; FLAP = 5-lipoxygenase-activating protein; g = grams; GST = glutathione S-transferases (gene); HR = hazard ratio; IGF = insulin-like growth factor; IQR: interquartile range; IRR: incidence rate ratio; mg = milligrams; MIXED = results were both in favor and not in favor of DHA or EPA intake or higher levels of DHA or EPA and risk of cancer depending on the genetic mutations or gene expression pathways; MPO = myeloperoxidase; MSI = microsatellite instability; NAT = Nacetyltransferases; n-3 LC-PUFA = omega-3 long-chain polyunsaturated fatty acids; NEG = results not in favor of DHA or EPA intake or higher levels of DHA or EPA and risk of cancer with certain genetic mutations or gene expression pathways; Nf-KB = Nuclear factor Kappa B; NR: not reported; NS = no statistically significant results; OGG1 = 8-Oxoguanine glycosylase; OR = odds ratio; PARP = poly ADP ribose polymerase (gene); PCA = prostate cancer; POS: results in favor of DHA or EPA intake or higher levels of DHA or EPA and risk of cancer with certain genetic mutations or gene expression pathways; PPL = plasma phospholipid(s); PR = progesterone receptor; PR+ = progesterone receptor positive; PR− = progesterone receptor negative; PTGS1 = prostaglandin-endoperoxide synthase 1 (gene); PTGS2 = prostaglandin-endoperoxide synthase 2 (gene); PUFA = polyunsaturated fatty acids; Q = quantile or quartile or quintile; RBC = red blood cell; RR = risk ratio; SNP = single nucleotide polymorphisms; tagSNP = a single nucleotide polymorphism used to tag a particular haplotype within the genome; TGF-β = transforming growth factor-beta; UGT = UDP Glucuronosyltransferase; XPD = XRCC1 = X-ray repair cross-complementing protein 1.

## References

[1] Ferlay J., Colombet M., Soerjomataram I., Mathers C., Parkin D.M., Piñeros M., Znaor A., Bray F. (2019). Estimating the global cancer incidence and mortality in 2018: GLOBOCAN sources and methods. Int. J. Cancer.

[2] Heron M. (2019). Deaths: Leading Causes for 2017.

[3] Triff K., Kim E., Chapkin R.S. (2015). Chemoprotective epigenetic mechanisms in a colorectal cancer model: Modulation by n-3 PUFA in combination with fermentable fiber. Curr. Pharm. Rep..

[4] Calder P.C. (2018). Very long-chain n-3 fatty acids and human health: Fact, fiction and the future. Proc. Nutr. Soc..

[5] Gao M., Sun K., Guo M., Gao H., Liu K., Yang C., Li S., Liu N. (2015). Fish consumption and n-3 polyunsaturated fatty acids, and risk of hepatocellular carcinoma: Systematic review and meta-analysis. Cancer Causes Control.

[6] Liu J., Abdelmagid S.A., Pinelli C.J., Monk J.M., Liddle D.M., Hillyer L.M., Hucik B., Silva A., Subedi S., Wood G.A. (2018). Marine fish oil is more potent than plant-based n-3 polyunsaturated fatty acids in the prevention of mammary tumors. J. Nutr. Biochem..

[7] Liu J., Ma D.W. (2014). The role of n-3 polyunsaturated fatty acids in the prevention and treatment of breast cancer. Nutrients.

[8] Mauermann J., Pouliot F., Fradet V. (2011). Dietary omega-3 fatty acids, genetic variation and risk of breast and prostate cancers. World Review of Nutrition and Dietetics.

[9] Serini S., Calviello G. (2017). Modulation of Ras/ERK and Phosphoinositide Signaling by Long-Chain n-3 PUFA in Breast Cancer and Their Potential Complementary Role in Combination with Targeted Drugs. Nutrients.

[10] Yang B., Wang F.L., Ren X.L., Li D. (2014). Biospecimen long-chain N-3 PUFA and risk of colorectal cancer: A meta-analysis of data from 60,627 individuals. PLoS ONE.

[11] Gago-Dominguez M., Castelao J.E., Sun C.L., Van Den Berg D., Koh W.P., Lee H.P., Yu M.C. (2004). Marine n-3 fatty acid intake, glutathione S-transferase polymorphisms and breast cancer risk in post-menopausal Chinese women in Singapore. Carcinogenesis.

[12] Newell M., Brun M., Field C.J. (2019). Treatment with DHA Modifies the Response of MDA-MB-231 Breast Cancer Cells and Tumors from nu/nu Mice to Doxorubicin through Apoptosis and Cell Cycle Arrest. J. Nutr..

[13] VanderSluis L., Mazurak V.C., Damaraju S., Field C.J. (2017). Determination of the Relative Efficacy of Eicosapentaenoic Acid and Docosahexaenoic Acid for Anti-Cancer Effects in Human Breast Cancer Models. Int. J. Mol. Sci..

[14] Corella D., Ordovas J.M. (2012). Interactions between dietary n-3 fatty acids and genetic variants and risk of disease. Br. J. Nutr..

[15] Fradet V., Cheng I., Casey G., Witte J.S. (2009). Dietary omega-3 fatty acids, cyclooxygenase-2 genetic variation, and aggressive prostate cancer risk. Clin. Cancer Res. Off. J. Am. Assoc. Cancer Res..

[16] Hedelin M., Chang E.T., Wiklund F., Bellocco R., Klint A., Adolfsson J., Shahedi K., Xu J., Adami H.O., Gronberg H. (2007). Association of frequent consumption of fatty fish with prostate cancer risk is modified by COX-2 polymorphism. Int. J. Cancer.

[17] Khankari N.K., Bradshaw P.T., Steck S.E., He K., Olshan A.F., Ahn J., Terry M.B., Crew K.D., Teitelbaum S.L., Neugut A.I. (2016). Interaction between polyunsaturated fatty acids and genetic variants in relation to breast cancer incidence. J. Cancer Epidemiol. Prev..

[18] Stern M.C., Siegmund K.D., Corral R., Haile R.W. (2005). XRCC1 and XRCC3 polymorphisms and their role as effect modifiers of unsaturated fatty acids and antioxidant intake on colorectal adenomas risk. Cancer Epidemiol. Biomark. Prev..

[19] Ceschi M., Sun C.L., Van Den Berg D., Koh W.P., Yu M.C., Probst-Hensch N. (2005). The effect of cyclin D1 (CCND1) G870A-polymorphism on breast cancer risk is modified by oxidative stress among Chinese women in Singapore. Carcinogenesis.

[20] Molfino A., Amabile M.I., Mazzucco S., Biolo G., Farcomeni A., Ramaccini C., Antonaroli S., Monti M., Muscaritoli M. (2017). Effect of oral docosahexaenoic acid (DHA) supplementation on DHA levels and omega-3 index in red blood cell membranes of breast cancer patients. Front. Physiol..

[21] Stripp C., Overvad K., Christensen J., Thomsen B.L., Olsen A., Moller S., Tjonneland A. (2003). Fish intake is positively associated with breast cancer incidence rate. J. Nutr..

[22] Bassett J.K., Hodge A.M., English D.R., MacInnis R.J., Giles G.G. (2016). Plasma phospholipids fatty acids, dietary fatty acids, and breast cancer risk. Cancer Causes Control.

[23] Kim E.H., Willett W.C., Colditz G.A., Hankinson S.E., Stampfer M.J., Hunter D.J., Rosner B., Holmes M.D. (2006). Dietary fat and risk of postmenopausal breast cancer in a 20-year follow-up. Am. J. Epidemiol..

[24] Kiyabu G.Y., Inoue M., Saito E., Abe S.K., Sawada N., Ishihara J., Iwasaki M., Yamaji T., Shimazu T., Sasazuki S. (2015). Fish, n3 polyunsaturated fatty acids and n6 polyunsaturated fatty acids intake and breast cancer risk: The Japan Public Health Center-based prospective study. Int. J. Cancer.

[25] Park S.Y., Kolonel L.N., Henderson B.E., Wilkens L.R. (2012). Dietary fat and breast cancer in postmenopausal women according to ethnicity and hormone receptor status: The Multiethnic Cohort Study. Cancer Prev. Res..

[26] Habermann N., Ulrich C.M., Lundgreen A., Makar K.W., Poole E.M., Caan B., Kulmacz R., Whitton J., Galbraith R., Potter J.D. (2013). PTGS1, PTGS2, ALOX5, ALOX12, ALOX15, and FLAP SNPs: Interaction with fatty acids in colon cancer and rectal cancer. Genes Nutr..

[27] Kantor E.D., Lampe J.W., Peters U., Vaughan T.L., White E. (2014). Long-chain omega-3 polyunsaturated fatty acid intake and risk of colorectal cancer. Nutr. Cancer.

[28] Song M., Nishihara R., Wu K., Qian Z.R., Kim S.A., Sukawa Y., Mima K., Inamura K., Masuda A., Yang J. (2015). Marine omega-3 polyunsaturated fatty acids and risk of colorectal cancer according to microsatellite instability. J. Natl. Cancer Inst..

[29] Song M., Nishihara R., Cao Y., Chun E., Qian Z.R., Mima K., Inamura K., Masugi Y., Nowak J.A., Nosho K. (2016). Marine omega-3 Polyunsaturated Fatty Acid Intake and Risk of Colorectal Cancer Characterized by Tumor-Infiltrating T Cells. JAMA Oncol..

[30] Stern M.C., Butler L.M., Corral R., Joshi A.D., Yuan J.M., Koh W.P., Yu M.C. (2009). Polyunsaturated fatty acids, DNA repair single nucleotide polymorphisms and colorectal cancer in the Singapore Chinese Health Study. J. Nutr. Nutr..

[31] Theodoratou E., Campbell H., Tenesa A., McNeill G., Cetnarskyj R., Barnetson R.A., Porteous M.E., Dunlop M.G., Farrington S.M. (2008). Modification of the associations between lifestyle, dietary factors and colorectal cancer risk by APC variants. Carcinogenesis.

[32] Volpato M., Perry S.L., Marston G., Ingram N., Cockbain A.J., Burghel H., Mann J., Lowes D., Wilson E., Droop A. (2016). Changes in plasma chemokine C-C motif ligand 2 levels during treatment with eicosapentaenoic acid predict outcome in patients undergoing surgery for colorectal cancer liver metastasis. Oncotarget.

[33] Andersen V., Holst R., Kopp T.I., Tjonneland A., Vogel U. (2013). Interactions between Diet, Lifestyle and IL10, IL1B, and PTGS2/COX-2 Gene Polymorphisms in Relation to Risk of Colorectal Cancer in a Prospective Danish Case-Cohort Study. PLoS ONE.

[34] Luchtenborg M., Weijenberg M.P., de Goeij A.F., Wark P.A., Brink M., Roemen G.M., Lentjes M.H., de Bruine A.P., Goldbohm R.A., van’t Veer P. (2005). Meat and fish consumption, APC gene mutations and hMLH1 expression in colon and rectal cancer: A prospective cohort study (The Netherlands). Cancer Causes Control.

[35] Slattery M.L., Curtin K., Wolff R.K., Herrick J.S., Caan B.J., Samowitz W. (2010). Diet, physical activity, and body size associations with rectal tumor mutations and epigenetic changes. Cancer Causes Control.

[36] Tiemersma E.W., Kampman E., Bueno de Mesquita H.B., Bunschoten A., van Schothorst E.M., Kok F.J., Kromhout D. (2002). Meat consumption, cigarette smoking, and genetic susceptibility in the etiology of colorectal cancer: Results from a Dutch prospective study. Cancer Causes Control.

[37] Almendingen K., Hostmark A.T., Fausa O., Mosdol A., Aabakken L., Vatn M.H. (2007). Familial adenomatous polyposis patients have high levels of arachidonic acid and docosahexaenoic acid and low levels of linoleic acid and alpha-linolenic acid in serum phospholipids. Int. J. Cancer.

[38] West N.J., Clark S.K., Phillips R.K., Hutchinson J.M., Leicester R.J., Belluzzi A., Hull M.A. (2010). Eicosapentaenoic acid reduces rectal polyp number and size in familial adenomatous polyposis. Gut.

[39] Chan J.M., Weinberg V., Magbanua M.J., Sosa E., Simko J., Shinohara K., Federman S., Mattie M., Hughes-Fulford M., Haqq C. (2011). Nutritional supplements, COX-2 and IGF-1 expression in men on active surveillance for prostate cancer. Cancer Causes Control.

[40] Magbanua M.J.M., Roy R., Sosa E.V., Weinberg V., Federman S., Mattie M.D., Hughes-Fulford M., Simko J., Shinohara K., Haqq C.M. (2011). Gene expression and biological pathways in tissue of men with prostate cancer in a randomized clinical trial of lycopene and fish oil supplementation. PLoS ONE.

[41] Cheng T.Y., King I.B., Barnett M.J., Ambrosone C.B., Thornquist M.D., Goodman G.E., Neuhouser M.L. (2013). Serum phospholipid fatty acids, genetic variation in myeloperoxidase, and prostate cancer risk in heavy smokers: A gene-nutrient interaction in the carotene and retinol efficacy trial. Am. J. Epidemiol..

[42] Cui T., Hester A.G., Seeds M.C., Rahbar E., Howard T.D., Sergeant S., Chilton F.H. (2016). Impact of Genetic and Epigenetic Variations Within the FADS Cluster on the Composition and Metabolism of Polyunsaturated Fatty Acids in Prostate Cancer. Prostate.

[43] Galet C., Gollapudi K., Stepanian S., Byrd J.B., Henning S.M., Grogan T., Elashoff D., Heber D., Said J., Cohen P. (2014). Effect of a low-fat fish oil diet on proinflammatory eicosanoids and cell-cycle progression score in men undergoing radical prostatectomy. Cancer Prev. Res..

[44] Khankari N.K., Murff H.J., Zeng C., Wen W., Eeles R.A., Easton D.F., Kote-Jarai Z., Al Olama A.A., Benlloch S., Muir K. (2016). Polyunsaturated fatty acids and prostate cancer risk: A Mendelian randomisation analysis from the PRACTICAL consortium. Br. J. Cancer.

[45] Catsburg C., Joshi A.D., Corral R., Lewinger J.P., Koo J., John E.M., Ingles S.A., Stern M.C. (2012). Polymorphisms in carcinogen metabolism enzymes, fish intake, and risk of prostate cancer. Carcinogenesis.

[46] Fahrmann J.F., Ballester O.F., Ballester G., Witte T.R., Salazar A.J., Kordusky B., Cowen K.G., Ion G., Primerano D.A., Boskovic G. (2013). Inhibition of nuclear factor kappa B activation in early-stage chronic lymphocytic leukemia by omega-3 fatty acids. Cancer Investig..

[47] Cury-Boaventura M.F., Torrinhas R.S.M.D.M., Godoy A.B.P.D., Curi R., Waitzberg D.L. (2012). Human leukocyte death after a preoperative infusion of medium/long-chain triglyceride and fish oil parenteral emulsions: A randomized study in gastrointestinal cancer patients. J. Parenter. Enter. Nutr..

[48] Morales E., Porta M., Vioque J., Lopez T., Mendez M.A., Pumarega J., Malats N., Crous-Bou M., Ngo J., Rifa J. (2007). Food and nutrient intakes and K-ras mutations in exocrine pancreatic cancer. J. Epidemiol. Community Health.

[49] Huang L., Yuan K., Liu J., Ren X., Dong X., Tian W., Jia Y. (2014). Polymorphisms of the TLR4 gene and risk of gastric cancer. Gene.

[50] Bernard-Gallon D.J., Vissac-Sabatier C., Antoine-Vincent D., Rio P.G., Maurizis J.C., Fustier P., Bignon Y.J. (2002). Differential effects of n-3 and n-6 polyunsaturated fatty acids on BRCA1 and BRCA2 gene expression in breast cell lines. Br. J. Nutr..

[51] Jourdan M.L., Mahéo K., Barascu A., Goupille C., De Latour M.P., Bougnoux P., Rio P.G. (2007). Increased BRCA1 protein in mammary tumours of rats fed marine omega-3 fatty acids. Oncol. Rep..

[52] Yang Y., Dong X., Xie B., Ding N., Chen J., Li Y., Zhang Q., Qu H., Fang X. (2015). Databases and web tools for cancer genomics study. Genom. Proteom. Bioinform..

[53] Cockbain A.J., Volpato M., Race A.D., Munarini A., Fazio C., Belluzzi A., Loadman P.M., Toogood G.J., Hull M.A. (2014). Anticolorectal cancer activity of the omega-3 polyunsaturated fatty acid eicosapentaenoic acid. Gut.

[54] Shao N., Feng N., Wang Y., Mi Y., Li T., Hua L. (2012). Systematic review and meta-analysis of COX-2 expression and polymorphisms in prostate cancer. Mol. Biol. Rep..

[55] Wang X.F., Huang M.Z., Zhang X.W., Hua R.X., Guo W.J. (2013). COX-2-765G>C polymorphism increases the risk of cancer: A meta-analysis. PLoS ONE.

[56] Hou T.Y., Davidson L.A., Kim E., Fan Y.Y., Fuentes N.R., Triff K., Chapkin R.S. (2016). Nutrient-Gene Interaction in Colon Cancer, from the Membrane to Cellular Physiology. Annu. Rev. Nutr..

[57] Godet I., Gilkes D. (2017). BRCA1 and BRCA2 mutations and treatment strategies for breast cancer. Integr. Cancer Sci. Ther..

[58] Zheng J.S., Hu X.J., Zhao Y.M., Yang J., Li D. (2013). Intake of fish and marine n-3 polyunsaturated fatty acids and risk of breast cancer: Meta-analysis of data from 21 independent prospective cohort studies. BMJ Clin. Res. Ed..

[59] Chen G.C., Qin L.Q., Lu D.B., Han T.M., Zheng Y., Xu G.Z., Wang X.H. (2015). N-3 polyunsaturated fatty acids intake and risk of colorectal cancer: Meta-analysis of prospective studies. Cancer Causes Control.

[60] Yu X.F., Zou J., Dong J. (2014). Fish consumption and risk of gastrointestinal cancers: A meta-analysis of cohort studies. World J. Gastroenterol..

[61] World Cancer Research Fund/American Institute for Cancer Research Diet, Nutrition, Physical Activity and Cancer: A Global Perspective.

[62] Kuratko C.N., Salem N. (2009). Biomarkers of DHA status. Prostaglandins Leukot. Essent. Fat. Acids.

[63] Munn Z., Peters M., Stern C., Tufanaru C., McArthur A., Aromataris E. (2018). Systematic review or scoping review? Guidance for authors when choosing between a systematic or scoping review approach. BMC Med. Res. Methodol..

[64] Sucharew H. (2019). Methods for Research Evidence Synthesis: The Scoping Review Approach. J. Hosp. Med..

[65] Hu Y., Hu F.B., Manson J.E. (2019). Marine Omega-3 Supplementation and Cardiovascular Disease: An Updated Meta-Analysis of 13 Randomized Controlled Trials Involving 127 477 Participants. J. Am. Heart Assoc..

[66] Schulze M.B., Martínez-González M.A., Fung T.T., Lichtenstein A.H., Forouhi N.G. (2018). Food based dietary patterns and chronic disease prevention. BMJ (Clin. Res. Ed.).

[67] Shim J.S., Oh K., Kim H.C. (2014). Dietary assessment methods in epidemiologic studies. Epidemiol. Health.

[68] Maki K.C., Slavin J.L., Rains T.M., Kris-Etherton P.M. (2014). Limitations of observational evidence: Implications for evidence-based dietary recommendations. Adv. Nutr..

